# The Functional and Radiological Outcome of Hoffa's Fracture Treated With Cannulated Cancellous Screws

**DOI:** 10.7759/cureus.23829

**Published:** 2022-04-04

**Authors:** Ajay Kurahatti, Hariprasad Seenappa, Arun H Shanthappa, Nagakumar J S

**Affiliations:** 1 Department of Orthopaedics, Sri Devaraj Urs Academy of Higher Education and Research, Kolar, IND

**Keywords:** cannulated cancellous screw, articular anatomy, lag screw, neers score, hoffa's fracture

## Abstract

Introduction: Distal femur AO type 33 B fractures consist of partial articular fractures subdivided into three types namely sagittal lateral condyle fracture, medial condyle fracture, and coronal split fracture. Coronal plane fractures of the distal femur are less frequent compared to sagittal plane fractures and are known as Hoffa fractures. The mechanism of injury is usually a direct anteroposterior force to the flexed and abducted knee for lateral condylar fractures and a direct impact on the medial side of the knee in flexion for a medial condylar fracture. Various approaches like lateral parapatellar for lateral condylar Hoffa's fixation, with or without posterior approach for open reduction of Hoffa's fracture with screw or buttress plate fixation, medial parapatellar approach for medial condylar Hoffa's fracture screw fixation are used.

Materials and Methods: This study was conducted at R L Jalappa Hospital and Research Center attached to Sri Devaraj Urs Medical College, Kolar, India, from June 2017 to May 2020 with 17 patients as a sample size.

Results: Seventeen patients with Hoffa's fracture were treated with cannulated cancellous screws with lag effect including 12 males and five females with a mean age of 31.1 years. The range of motion ranged from 120 to 135 degrees of flexion with a mean of 125.2 degrees. Three patients had extensor lag ranging from 5 to 10 degrees with an average of 6.3 degrees. Neer scores were excellent in 11, good in four, and fair in two patients. The average fracture union time for the lateral condyle was 16.4 months and for the medial condyle, it was 16.7 months.

Conclusion: Restoration of articular anatomy and its congruence is of paramount importance for better surgical outcomes. Closed or open reduction and stable fixation with anteroposterior cannulated cancellous screws are essential. A good post-operative rehabilitation program is required for better outcomes.

## Introduction

Distal femur AO type 33 B fractures comprise partial articular fractures subdivided into three types namely sagittal lateral condyle fracture, medial condyle fracture, and coronal split fracture. Coronal plane fractures of the distal femur are less frequent compared to sagittal plane fractures and were described by Hoffa in 1904 and are known as Hoffa fractures (AO type B3). Hoffa's fracture is a tangential, unicondylar fracture of the distal femoral condyle and is a relatively rare injury. They are isolated fractures of the femoral condyle and rare in occurrence [1]. Lateral condyle Hoffa fractures are three times more common than medial condyle fractures [2]. Hoffa's fracture and type 33 B fractures may affect either of the condyles but they have a preponderance to affect the lateral condyle due to physiological valgus and the direction of force, which is usually direct trauma to flexed knee with an element of slight abduction. Hoffa's fractures are usually missed on a plain radiograph in approximately 30% of cases and the incidence of these fractures in type 33 B fractures is 38% [1].

Fracture of the distal femur depends on the velocity of injury or the amount of osteoporosis involved. It follows a zone of weakness in the anatomic location of the distal femur, which leads to a fracture in a specific pattern. These are the transition from diaphysis to the metaphysis, intercondylar notch where the patella acts as a wedge and area between trochlear groove and medial or lateral condyle. The distal femur in the cross-section is trapezoid and anterior and posterior surfaces are not parallel to each other. Also, there is an inclination of 10 degrees on the medial and 25 degrees on the lateral aspect [1].

The mechanism of injury has been reported to be a direct anteroposterior force to the flexed and abducted knee for lateral condylar fractures and a direct impact on the medial side of the knee in flexion for a medial condylar fracture. The combinations of forces including vertical thrust and twisting may bring about this intra-articular fracture of the knee. This fracture is intrinsically unstable owing to bony instability and muscular pull [3].

Hoffa's fractures are further classified according to Letenneur into three types: Type I - Fracture line is parallel to the posterior femoral cortex involving the entire posterior condyle; Type II - Fracture occurs in the area behind the line parallel to the posterior femoral cortex; Type III - Fracture line runs obliquely, therefore, responds poorly to conservative treatment.

The conservative management yields poor results and, hence, firm internal fixation is the treatment of choice for this fracture. Nonoperative treatment in these fractures usually fails due to intra-articular involvement, multi-fragmentary morphology, and approaches to treat type 33 B fractures.

Surgical approaches to these fractures depend upon the condyle involved, the location of the fracture line, and also the presence of comminution [1]. Several articles have been published documenting superior functional results using internal fixation [4,5,6]. Various approaches like lateral parapatellar for lateral condylar Hoffa's fixation, with or without posterior approach for open reduction of Hoffa's fracture with screw or buttress plate fixation, medial parapatellar for medial condylar Hoffa's fracture screw fixation are used. Another is the swashbuckler approach, which is a modified anterior approach and provides improved exposure and spares quadriceps muscle bellies. Also, the surgical scar does not interfere with subsequent total knee arthroplasty if required at a later date. It is useful for the fixation of distal femoral fractures with intra-articular involvement [1].

Nowadays, minimally invasive surgical stabilization is preferred as it preserves the bone biology while fixation. It is achieved by preoperative planning by tracing the fractured fragments and planning the incisions on the table. Under the fluoroscopic guidance, the different incisions are made for distal fixation and proximal fixation of screws. The ultimate goal is to be minimally invasive and also get appropriate alignment [1].

Two 6.5mm partially threaded cannulated cancellous screws are the preferred modality, especially in cases of undisplaced Hoffa's fracture to provide rotational stability. A medial parapatellar arthrotomy or a direct lateral approach between the iliotibial band and the biceps tendon may be required to expose the fracture [1]. Rigid fixation has also enabled earlier knee motion and weight-bearing, which help prevent some of the serious complications attributed to prolonged bed rest and traction [7,8].

A broad condylar buttress plate may be used as a treatment modality for displaced Hoffa's fracture associated with osteoporosis or associated with the supracondylar component. Articular reductions were classified as anatomical acceptable (<2 mm step) and poor (>2 mm) on the immediate post-operative radiographs [1].

All techniques have their own merits and demerits and still more studies are required to conclude which will be the better modality of treatment for a particular fracture pattern. We conducted this study to analyze the clinico-radiological and functional outcomes along with complications of surgically managed isolated Hoffa fracture with a cannulated cancellous screw.

## Materials and methods

This study was conducted at R.L. Jalappa Hospital and Research Center attached to Sri Devaraj Urs Medical College, Kolar, India from June 2017 to May 2020 with 17 patients as a sample size. The sample size was estimated based on Neer's knee evaluation scores with an average variance of 1.96 from a study conducted in 2017 [9]. To detect a difference of 9.5% difference in mean Neer's knee evaluation scores with 80% power and with a 95% confidence interval, the sample size required was 15 cases. As a dropout rate of 10% was expected during follow-up, the final sample size was estimated as 15+2=17. This study protocol was reviewed and approved by the Institutional Ethics Committee of Sri Devaraj Urs Medical College, Kolar, India (DMC/KLR/IEC/396/2020-21). This study was carried out in strict accordance with the Declaration of Helsinki and its amendments. Informed consent was obtained from all the study participants.

The patients included were adults with undisplaced and displaced Hoffa's fracture, open fractures type I, type II, type IIIA Gustilo-Anderson classification of distal femur fractures, polytrauma patients. Patients with pathological fractures, non-union, associated neurovascular injury, and crush injuries were excluded.

Preoperative radiographs and routine surgical profile of blood investigations were done for all study patients and computerized tomography (CT) scan was done for comminuted Hoffa's fracture patients. Then the patients were operated on after informed written consent. Post-operatively, the patients were followed up at six weeks, three months, six months, and one year as documented in the inpatient and outpatient records of the patients. Patients were immobilized with a long leg knee brace for three weeks with non-weight-bearing walker-assisted walking and static quadriceps exercises. Patients were allowed a progressive active knee range of motion (ROM) as tolerated and assisted knee ROM from the fourth week. Partial weight-bearing was allowed from six weeks to full weight-bearing by twelve weeks.

The data were statistically evaluated with IBM SPSS Statistics for Windows, Version 20.0 (Released 2011; IBM Corp., Armonk, New York, United States).

## Results

Seventeen patients with Hoffa's fracture were treated with cannulated cancellous screws with lag effect including 12 males and five females aged from 24 years to 47 years with a mean age of 31.1 years. Four patients sustained falls from height and 13 patients had road traffic accidents as described in Table 1. Right-sided femurs were involved in nine patients and eight patients had left-sided femur fractures. Of the patients, 16 sustained closed fractures and one had an open fracture, as depicted in Table 1. Among the 17 patients, according to Letenneur classification, lateral femoral condyle fractures include three of type I, four of type II, and two of type III; medial femoral condyle fractures include three of type I, three of type II, and two of type III. All the patients were followed up for a duration of 12 to 22 months with a mean duration of 17 months. There were no complications like injury to a popliteal artery or tibial or peroneal nerve.

**Table 1:** Mechanism of injury and type of fracture

	Number of patients (%)
Mechanism of injury	
Fall from height	4 (24%)
Road traffic accident	13 (76%)
Type of fracture	
Closed	16 (94%)
Open	1 (6%)

**Figure 1:**
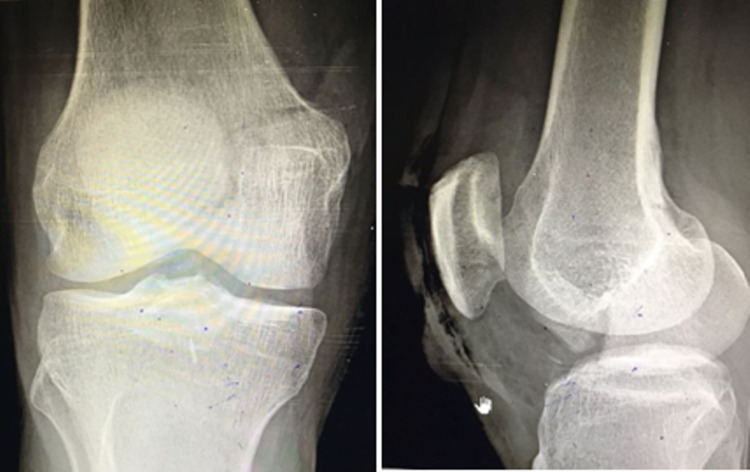
Anteroposterior and lateral radiograph of medial condyle Hoffa's fracture before surgery

Figure 1 shows anteroposterior and lateral radiograph of medial condyle Hoffa's fracture before surgery.

**Figure 2:**
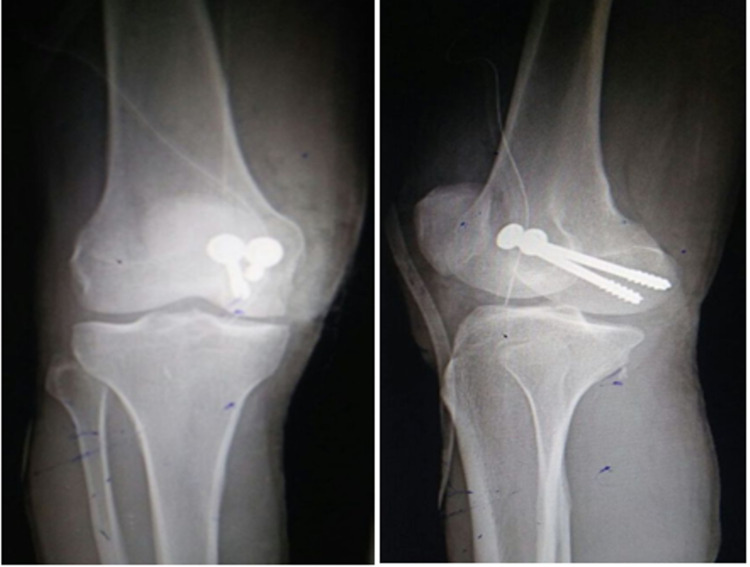
Anteroposterior and lateral radiograph of medial condyle Hoffa's fracture after fixation.

Figure 2 shows antero-posterior and lateral radiograph after fracture fixation, which healed in further weeks.

The ROM ranged from 120 to 135 degrees of flexion with a mean of 125.2 degrees as described in Table 2. Table 3 depicts the percentage of fractures getting united at different time periods. Average fracture union time for lateral condyle was 16.4 months and for medial condyle was 16.7 months. Three patients had extensor lag ranging from 5-10 degrees with an average of 6.3 degrees. Neer scores were excellent in 11 patients, good in four patients, and fair in two patients as described in Table 4. There was no subluxation or dislocation of the knee joint noted.

**Table 2:** Range of flexion

Range of flexion (Degrees)	Number of patients	Percentage
120-130	15	88
131-140	2	12

**Table 3:** Time of union

Union (weeks)	Number of patients	Percentage
12-13	2	12
14-15	6	35
16-17	7	41
18-20	2	12

**Table 4:** Neer's scores

Neer Scores	Number of patients	Percentage
Excellent (>85)	11	65
Good (70-84)	4	23
Fair (50-69)	2	12
Poor (<50)	0	0

**Figure 3:**
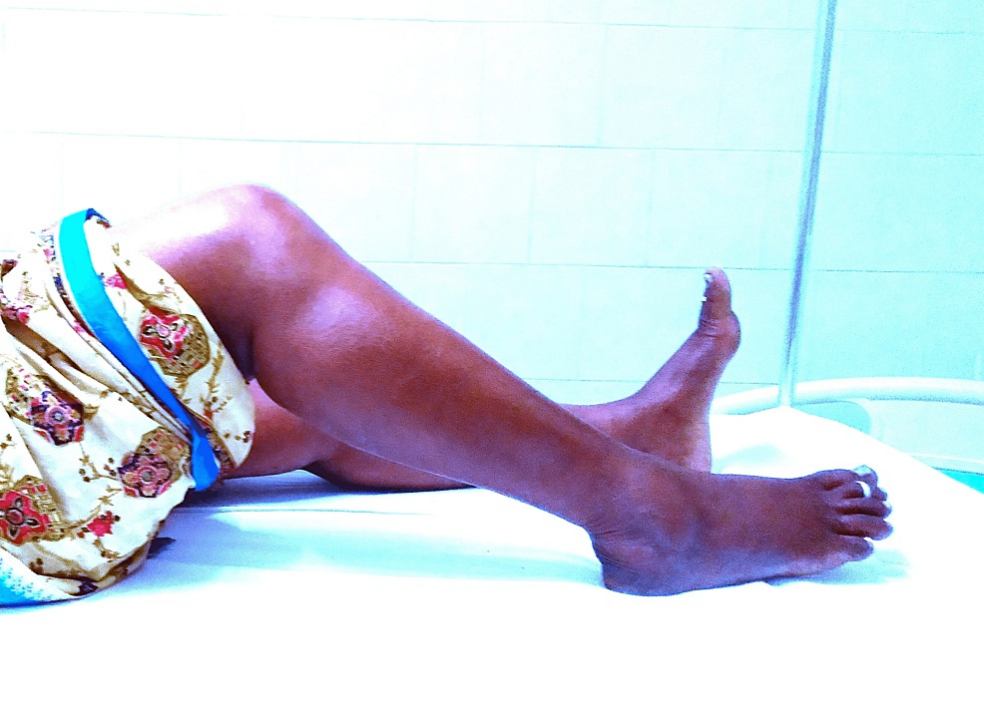
Post-operative follow-up of the patient with range of motion of knee at six weeks

**Figure 4:**
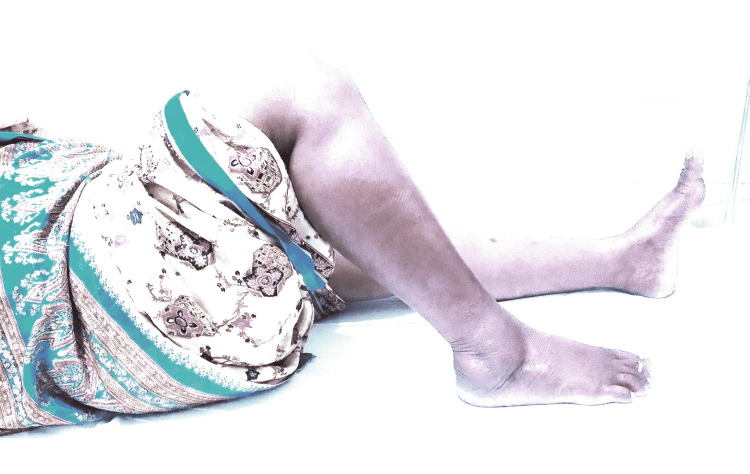
Post-operative follow-up of a patient with range of motion of knee at six months

**Figure 5:**
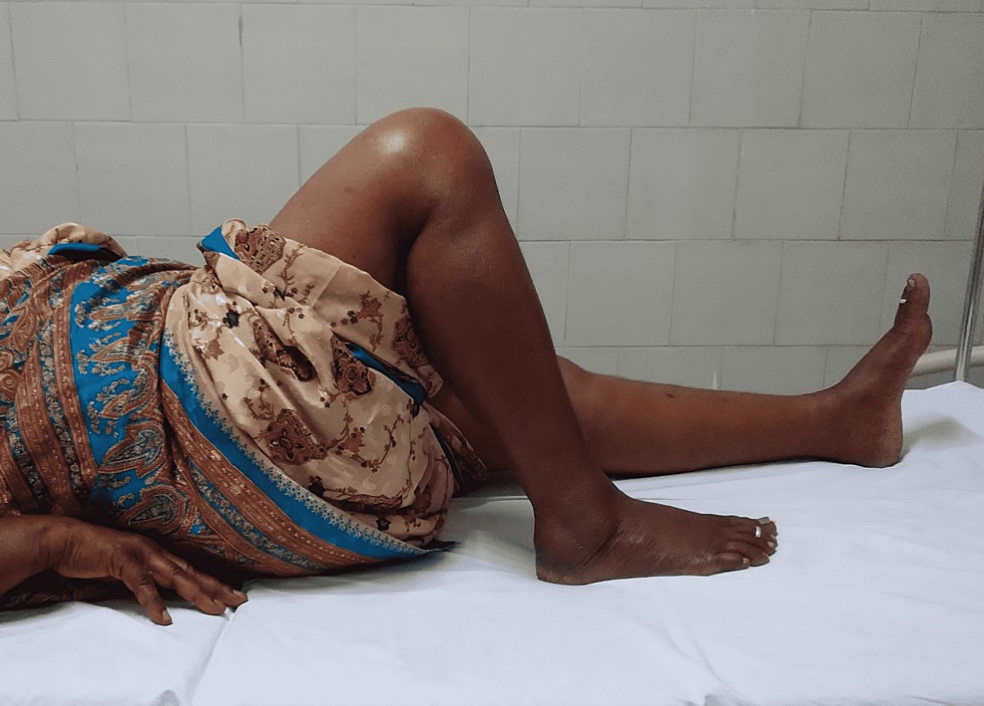
Post-operative follow-up of a patient with range of motion of knee at one year

Figure 3, Figure 4, and Figure 5 show the post-operative follow-ups of a patient with ROM of the knee at six weeks, six months, and one year, respectively.

Most of the patients were treated with 6.5-mm (four patients) or 4-mm (seven patients) partially threaded cannulated cancellous screws in an anteroposterior direction in lag mode. The mean tourniquet time was 78.5 +/- 10.68 min and the mean blood loss was found to be 200 ml. All the patients underwent surgery after a mean delay of 2.02+/- 1.06 days after injury. Table 5 describes the details of all the patients.

**Table 5:** Patient details L: left; r: right; LC: lateral condyle; MC: medial condyle; M: male; F: female; ROM: range of motion

No.	Age/sex	Side/site	Fixation	Neer's score	ROM	Complications
1	25/M	L/MC	AP screw/4 mm	87	0-120	
2	30/F	L/LC	AP screw/6.5 mm	80	0-125	
3	41/M	R/LC	AP screw/4 mm	90	0-125	
4	25/M	R/MC	AP screw/6.5 mm	89	0-130	Surgical site infection
5	30/M	L/LC	AP screw/4 mm	88	0-125	
6	39/M	R/MC	AP screw/4 mm	83	0-120	
7	33/F	R/LC	AP screw/4 mm	93	5-125	Extensor lag with stiffness
8	36/M	L/LC	AP screw/6.5 mm	91	0-135	
9	34/M	R/MC	AP screw/4 mm	78	0-125	
10	29/F	L/LC	AP screw/4 mm	92	0-120	
11	35/M	R/MC	AP screw/4 mm	68	0-125	
12	47/M	R/LC	AP screw/6.5 mm	91	0-135	
13	35/F	L/MC	AP screw/4 mm	84	10-125	Extensor lag with stiffness
14	36/M	R/MC	AP screw/6.5 mm	86	0-130	
15	30/M	L/LC	AP screw/4 mm	65	0-120	
16	24/F	R/LC	AP screw/4 mm	91	5-125	Extensor lag with stiffness.
17	35/M	L/MC	AP screw/6.5 mm	90	0-120	

## Discussion

Isolated Hoffa fracture is an infrequent injury and commonly emerges from the lateral femoral condyle in comparison to the medial condyle. There is no certainty about the exact mechanism leading to this injury. These fractures commonly occur following motor vehicle accidents [6,10].

The mechanism of injury continues to remain elusive. The usual mechanism is postulated to be a combination of vertical shearing and twisting forces [11]. Lewis et al. argued that with the knee flexed to just beyond 90 degrees, the lateral femoral condyle is the leading part of the knee to receive an oblique or lateral impact [10]. Direct trauma to this area, possibly with an element of abduction, results in the typical Hoffa fracture. The physiological genu valgum may be the underlying basis for the predominant lateral condyle involvement in these fractures [12].

Conservative management is generally associated with suboptimal results; therefore, management has evolved into open reduction and internal fixation at present, which is stemmed from the fact that like any intra-articular fractures, anatomic reduction and stable fixation are cornerstones for optimal outcome. However, no clear and straightforward dependable treatment rationale has been formulated due to the dearth of specific information in the literature. Substantial debate is going on concerning the surgical approach and fixation methods [9].

We used the anterior approach with lateral parapatellar arthrotomy for lateral Hoffa's fracture and the medial parapatellar approach for medial Hoffa's fracture. We ascertained that these approaches yielded ample visualization obligatory for optimum reduction and fixation with multiple cannulated cancellous screws. A specific approach to a particular Hoffa fracture gives sufficient direct exposure to the fracture, which also minimizes injury to the extensor mechanism. Hak et al. undertook the biomechanical assessment of internal fixation constructs using 3.5mm and 6.5mm screws and their data indicated that a double 6.5mm screw provided a meaningfully firm construct and at least two 3.5mm screws should be used to approximate the biomechanical stability of a single 6.5mm screw [13]. Jarit et al. and Arastu et al. show that fixation with a posteroanterior-oriented lag screw is biomechanically superior compared to that with an anteroposterior-oriented screw [14,15].

Arthroscopic-assisted reduction and internal fixation of femoral condyle have also appeared in recent times citing advantages of reduced soft tissue dissection, blood loss, operative time, and a faster recovery time [16]. However, no long-term trial exists comparing the outcomes of arthroscopic and open procedures. Whatever approach is used as per the surgeon’s preferences, adequate visualization of fracture fragments is paramount to aid the congruous reduction of articular surfaces and, hence, optimize the outcome [9].

Open reduction and internal fixation is the dominant treatment strategy for Hoffa fractures and has yielded satisfactory results in appropriate time periods. A variety of techniques and equipment have been discussed in the literature, most of which refer to the treatment of distal femur fractures. The surgical approach relies on the location of the injury and the presence or absence of posterior comminution [17].

For Letenneur II and some Letenneur III fractures close to the posterior cortex of the femoral condyle, cannulated lag screw fixation is commonly used. The exposed fracture line is initially fixed with a Kirschner wire (K-wire) and screws are placed perpendicular to the fracture surface. The use of several 3.5-mm-diameter screws is recommended to fix the fractures [18].

The average age of patients in our study was 31.1 years, which was comparable with Trikha et al. [9] where it was 34.8 years, Siddiqui et al. [19] showed 39.3 degrees and Singh et al. [20] showed 39.2 years. Males were more commonly affected in our series compared to females with 12:5 (70.58%:29.42%). In the study by Trikha et al., 25 males (78.12%) and seven females (21.88%) were affected [9]. In Siddiqui et al., 13 were males (81%) and three were females (19%) [19]. Singh et al.'s study show six males (75%) and two females (25 %) [20].

The mean ROM of the patients in our study was 125.2 degrees, which was comparably better than that of patients studied by Trikha et al. where it was 115 degrees [9]. Siddiqui et al.'s study shows 102.8 degrees [19], but was comparable with Singh et al. [20], which showed it as 115 degrees. The mean fracture union time for Hoffa's fracture for lateral condyle was 16.4 weeks and for the medial condyle was 16.7 weeks in our series compared to the average union time of 11.56 +/- 1.5 weeks as studied in a series of patients by Trikha et al. [9]. In a study by Siddiqui et al. [19], the average fracture union time was 15.5 weeks, but Singh et al. [20] reported fracture union time as 16 weeks. The Neer knee scores were excellent in 11 patients, good in four patients, and fair in two patients, which is comparable to a study by Siddiqui et al. [19].

Limitations of our study include being a retrospective study and having a small sample size. There was no comparative group; hence, it is difficult to determine or predict whether this surgical strategy is a better mode of treatment. As it was a retrospective study and post-operative CT was not done, the CT images were not available. Minimal invasive surgery is possible but it has technical challenges in patients having associated distal femur fractures and, hence, considerable experience is required for treatment of Hoffa's fractures.

## Conclusions

Hoffa fractures occur uncommonly and may be associated with extra-articular fractures, which require evaluation and an adequate plan of treatment. Restoration of articular anatomy and its congruence is of paramount importance for better surgical outcomes. Closed or open reduction and stable fixation with anteroposterior cannulated cancellous screws are essential. A good post-operative rehabilitation program is required for better outcomes.
